# Acute respiratory and ocular effects of electronic cigarettes: A pre–post experimental study in healthy users

**DOI:** 10.18332/tpc/216112

**Published:** 2026-03-10

**Authors:** Mercedes Segura Romero, Ignacio Garcia-Basterra, Sara Sánchez Martin, Jose Carlos Calero Valera, Alvaro Martinez Mesa, Javier Lopez Garcia, Miguel Benítez Cano-Gamonoso, Isabel Asschert Agüero, Jose Luis Velasco-Garrido, Eva Cabrera Cesar

**Affiliations:** 1 Department of Pneumology, Virgen de la Victoria University Hospital, Malaga, Spain; 2 Department of Ophthalmology, Virgen de la Victoria University Hospital, Malaga, Spain

**Keywords:** exhaled nitric oxide, pulmonary function tests, respiratory impedance, optical coherence tomography, retinal hemodynamics

## Abstract

**INTRODUCTION:**

The use of electronic cigarettes (ECs) has increased alarmingly among young people, often perceived as a safer alternative to conventional tobacco. Studies demonstrating that these are not harmless products are essential to raise awareness of their potential health risks.

**METHODS:**

A pre–post exposure experimental study was conducted between March and June 2024 at Virgen de la Victoria University Hospital, Málaga, Spain, in 40 healthy regular EC users aged ≥18 years. Respiratory parameters (FeNO, spirometry, oscillometry) and ophthalmological assessments (Schirmer test, Optical Coherence Tomography, and Pentacam) were evaluated before and after acute EC exposure (40–60 inhalations).

**RESULTS:**

Following acute exposure, a statistically significant decrease in FeNO was observed from 21.88 ± 22.01 to 18.05 ± 18.08 ppb (p=0.019), particularly in non-smokers (p=0.003), suggesting an airway inflammatory response. In dual users, a significant increase in pulmonary resistance from 0.21 ± 0.36 to 0.35 ± 0.48 kPa/L∙s (p=0.028) was detected. At the ocular level, the Schirmer test showed reduced tear secretion in 47.5% of participants. Additionally, significant changes were found in macular flow density (p=0.024) and inferior macular choroidal thickness (p=0.015), suggesting a possible retinal hemodynamic alteration.

**CONCLUSIONS:**

Acute use of ECs induces measurable changes in both respiratory function and ocular parameters. These findings underscore the need to raise awareness about the potential adverse effects of vaping and highlight the importance of further research into its long-term consequences.

## INTRODUCTION

The use of electronic cigarettes (ECs), has grown exponentially over the past decade, particularly among young people. In Europe, among adolescents aged 15–16 years, the prevalence of vaping ranges from 5.5% to 41%, with up to 20.8% reporting current use in some countries^1^. In Spain, one in four EC users had never smoked conventional tobacco, highlighting a possible gateway effect to nicotine addiction^1^. Although initially promoted as an alternative to conventional smoking, several studies have demonstrated the potential systemic adverse effects of ECs^2,3^. A recent systematic review reports that ECs may cause airway irritation, coughing, nausea, hemodynamic alterations, and endothelial inflammation^4^.

The composition of EC aerosols often includes nicotine, propylene glycol, vegetable glycerin, and multiple chemical substances such as aldehydes, heavy metals, and volatile organic compounds, many of which are irritant, pro-inflammatory, or potentially toxic^4^. Several studies have linked EC use to alterations in lung function, respiratory symptoms, and e-cigarette use^5-7^. Although fewer studies have addressed ocular involvement, there is emerging evidence that ECs can cause eye-related alterations^8^. Aldehydes and free radicals present in ECs may impair tear film stability, and vaping flavors may damage the lipid layer through lipid peroxidation^8^. These findings support the hypothesis of ocular disturbances associated with EC use, although larger cohorts and controlled studies are still needed.

The aim of this study was to evaluate the acute impact of EC use on respiratory function and ophthalmological parameters in healthy young individuals, using an experimental pre–post exposure design.

## METHODS

### Study design and population

An experimental pre–post study was conducted in a cohort of 40 healthy volunteers (absence of chronic respiratory, cardiovascular, or ocular disease) who were regular users of electronic cigarettes (ECs) (consumption for ≥1 month prior to recruitment). Data were collected between April and July 2024 at the Virgen de la Victoria University Hospital, Málaga, Spain. Male and female participants aged ≥18 years who had been using ECs for at least one month were included. Exclusion criteria were age <18 years, pregnancy, and known respiratory or ocular diseases.

### Procedure

The study was designed as a pre–post longitudinal assessment in a cohort of 40 healthy, habitual EC users. In the initial phase, each participant underwent a pulmonary evaluation including forced spirometry, fractional exhaled nitric oxide (FeNO) measurement, and impulse oscillometry. This was followed by an ophthalmological assessment, consisting of optical coherence tomography (OCT), anterior chamber analysis with Pentacam, and the Schirmer test to evaluate tear production.

After this baseline evaluation, participants used their own electronic cigarette device over a period of 40 to 60 minutes, with an estimated total of 40 to 60 puffs per subject. Upon completion of the exposure, all respiratory and ophthalmological tests were repeated to assess for potential changes induced by acute EC use.

### Ethical considerations

This study was conducted in accordance with the principles of the Declaration of Helsinki and Good Clinical Practice guidelines. Personal data were processed in compliance with Regulation (EU) 2016/679 of the European Parliament and of the Council of 27 April 2016 on the protection of natural persons with regard to the processing of personal data and on the free movement of such data, and with Spanish Organic Law 3/2018 of 5 December on the Protection of Personal Data and the Guarantee of Digital Rights. The study received approval from the Provincial Research Ethics Committee of Málaga, which deemed the project ethically and methodologically appropriate (approval code: ECEO-2023) on 14 March 2024.

### Statistical analysis

Statistical analysis was performed using SPSS software (IBM SPSS Statistics, version 29). Demographic variables (age, sex), tobacco use (exclusive EC use vs dual use), and device characteristics (nicotine-containing vs nicotine-free) were collected using structured questionnaires. Pulmonary parameters included FeNO (ppb), FEV1 (L and % predicted), FVC (L and % predicted), FEV1/FVC ratio, and oscillometry measures: AX (kPa/L) and R5–R20 (kPa/L∙s). Ophthalmological variables comprised Schirmer test results (mm/5 min), macular flow density (%), ganglion cell layer thickness (μm), retinal nerve fiber layer thickness (μm), and inferior macular choroidal thickness (μm).

Previously compiled databases for pulmonary and ocular parameters were imported for analysis. Quantitative variables were described using mean and standard deviation (SD), while qualitative variables were expressed as frequencies and percentages.

To compare quantitative variables before and after EC exposure, the paired Student’s t-test was used. For qualitative variables, the McNemar test was applied to detect significant differences between pre- and post-exposure measurements. Subgroup analyses were performed for exclusive EC users versus dual users to evaluate potential differential effects of nicotine or concurrent tobacco use. A p<0.05 was considered statistically significant.

## RESULTS

A total of 40 subjects were included, with a mean age of 23.75 ± 2.94 years; 30% were male (n=12) and 70% female (n=28). Among the participants, 60% (n=24) used nicotine-containing electronic cigarettes, while 40% (n=16) used nicotine-free devices. Additionally, 37.5% (n=15) were dual users (both tobacco and ECs), whereas 62.5% (n=25) did not smoke conventional tobacco.

### Respiratory results

Following acute EC exposure (40–60 inhalations), a statistically significant reduction in FeNO was observed, decreasing from 21.88 ± 22.01 to 18.05 ± 18.08 ppb (p=0.019). No significant changes were detected in spirometric values (FEV1, FVC, FEV1/FVC) or oscillometry parameters in the total sample (Table 1). Although the reductions observed in FEV1 and FVC were not statistically significant, a downward trend was noted across these parameters after exposure. However, subgroup analysis revealed that in dual users (n=15), a significant increase in peripheral airway resistance (R5–R20) was observed, rising from 0.21 ± 0.36 to 0.35 ± 0.48 kPa/L∙s (p=0.028). In exclusive EC users (n=25), this resistance remained unchanged (p=0.935), but a significant reduction in FeNO was found from 17.56 ± 13.73 to 13.52 ± 8.99 ppb (p=0.003) (Table 1).

**Table 1:** Comparison of respiratory parameters before and after acute electronic cigarette exposure in healthy adult volunteers (N=40)

*Variable*	*Pre-exposure* *Mean ± SD*	*Post-exposure* *Mean ± SD*	*p*
FeNO (ppb)	21.88 ± 22.014	18.05 ± 18.088	**0.019**
FEV1 (L)	3.61 ± 0.601	3.605 ± 0.63	0.682
FEV1 (% predicted)	92.60 ± 11.39	91.14 ± 12.08	0.159
FEV1/FCV ratio	0.84 ± 0.05	0.83 ± 0.07	0.110
FVC (L)	4.32 ± 0.75	4.35 ± 0.76	0.112
FVC (% predicted)	95.35 ± 11.67	96.43 ±11.31	0.110
Oscillometry: AX (kPa/L)	-0.124 ± 0.27	-0.145 ± 0.246	0.649
Oscillometry: R5–R20 (kPa/L∙s)	0.115 ± 0.34	0.166 ± 0.40	0.170

FeNO: fractional exhaled nitric oxide. FEV1: forced expiratory volume in 1 second. FVC: forced vital capacity. AX: area of reactance (kPa/L). R5–R20: difference between respiratory resistance at 5 Hz and 20 Hz (kPa/L∙s). P-values correspond to paired Student’s t-tests comparing pre- and post-exposure values.

### Ophthalmological results

**Figure 1:**
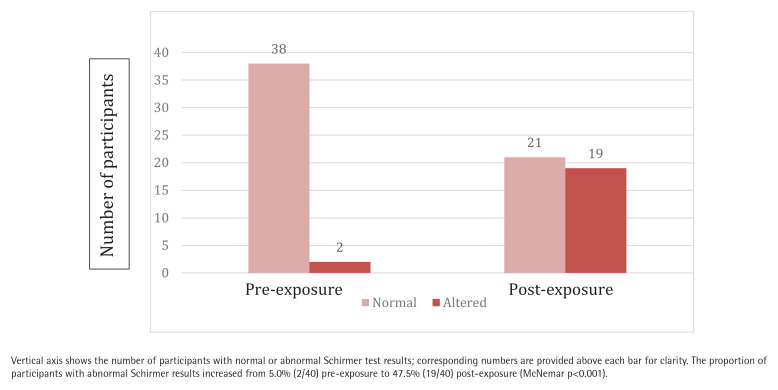
Schirmer test results before and after acute electronic cigarette exposure (N=40)

In the ophthalmological assessment, a significant functional impairment was observed following acute EC exposure. According to the McNemar test, the proportion of subjects with abnormal Schirmer results increased significantly from 5% pre-exposure to 47.5% post-exposure (p<0.001) (Figure 1).

Regarding the structural measurements obtained through OCT and Pentacam, a significant reduction in average macular flow density was observed after vaping (43.82 ± 2.01% vs 42.81± 3.03%; p=0.024). Similarly, a decrease in inferior macular choroidal thickness was detected (270.10 ± 69.62 vs 263.50 ± 64.71 μm; p=0.015). Other structural parameters, such as ganglion cell layer thickness and retinal nerve fiber layer (RNFL), did not show statistically significant changes, although a downward trend was observed following exposure (Table 2).

**Table 2:** Comparison of ophthalmological parameters before and after acute electronic cigarette exposure in healthy adult volunteers (N=40)

*Parameter*	*Pre-exposure* *Mean*	*Post-exposure* *Mean*	*p*
Average macular thickness (μm)	264.37	264.85	0.61
Average ganglion cell layer thickness (μm)	111.72	107.38	0.185
Average retinal nerve fiber layer (RNFL) thickness (μm)	107.83	105.93	0.101
Average macular flow density (%)	43.82	42.81	0.024
Schirmer test: number of subjects with abnormal result	2	19	-

P-values correspond to paired Student’s t-tests comparing pre- and post-exposure values. Schirmer test: abnormal result defined as ≤10 mm of wetting in 5 minutes. The change in the proportion of subjects with abnormal Schirmer results (from 5.0% to 47.5%) was analyzed using McNemar’s test (p<0.001).

## DISCUSSION

The results of this experimental study demonstrate that a single acute exposure to electronic cigarettes (ECs) can induce objectively measurable, short-term physiological alterations in both respiratory function and ophthalmological parameters in healthy young adults. This finding is particularly relevant from a public health perspective, as it highlights the potential risks associated with the use of these devices, which are often perceived by the general population, especially the youth, as a less harmful alternative to conventional tobacco.

At the respiratory level, a significant reduction in FeNO levels was identified, particularly among non-smokers. FeNO is a validated biomarker of eosinophilic airway inflammation, and its acute decrease may reflect respiratory epithelial dysfunction, oxidative stress, and reduced nitric oxide synthase (NOS) activity^9^. Previous studies, such as that by Kharitonov et al.^10^ have shown that conventional cigarette smokers have lower baseline FeNO levels, which further decrease after smoke inhalation. Similar findings have been reported in studies evaluating FeNO in EC users, also showing a reduction in FeNO levels^11^. In the case of ECs, Traboulsi et al.^12^ suggest that exposure to aerosols may induce neutrophilic inflammation, epithelial disruption, and the release of reactive oxygen species, leading to a subsequent decrease in FeNO^10^.

The absence of significant changes in spirometric parameters (FEV1, FVC, FEV1/FVC) may be explained by the single and acute nature of the exposure, as well as the young age and good baseline health of the participants. However, a trend toward decreased values was observed, which could become clinically relevant if exposure were repeated or chronic. This pattern has also been described in longitudinal studies of adolescent EC users, where progressive functional impairment compatible with mild airway obstruction has been documented^13^.

Oscillometry, a more sensitive tool for detecting alterations in peripheral pulmonary mechanics, revealed a significant increase in R5–R20 resistance in dual users in our study^14^. This finding suggests that the coexistence of smoking and vaping potentiates the acute inflammatory or bronchoconstrictive effect in an additive or synergistic manner. This pattern is consistent with the findings of Vardavas et al.^15^ who demonstrated increased respiratory resistance following brief EC use in healthy individuals. Subsequent studies, such as that by Antoniewicz et al.^14^, confirmed that even acute exposure to nicotine-containing EC aerosol can increase small airway resistance, as measured by impulse oscillometry. Similarly, other studies have reported increased airway resistance after single inhalations, with rises in R5 and R5–R20, supporting the utility of this technique in detecting subtle changes not captured by spirometry^16^. In contrast, no significant changes in pulmonary resistance were observed in exclusive EC users in our study, which may indicate a greater susceptibility to functional alterations in the context of dual use. These findings suggest a potential effect modification pattern, with conventional cigarette smoking acting as a moderator that amplifies the acute functional impact of EC exposure, as reflected by the greater increase in small-airway resistance observed in dual users compared with exclusive EC users.

Regarding ocular effects, this study adds clinical evidence suggesting that film dysfunction and retinal hemodynamic alterations following EC use. A reduction in Schirmer test results was observed in 47.5% of participants after acute exposure, reflecting significant tear film dysfunction. This is not merely a physiological alteration but may have serious consequences: tear deficiency can lead to tear film instability and tachykinetic lacrimation, conditions that promote ocular surface diseases such as keratitis and increase the risk of infections or corneal ulcers^17^. Moreover, persistent dryness raises tear osmolarity and triggers an inflammatory cascade in the ocular epithelium, potentially resulting in epithelial damage and neovascularization^18^. Wieslander et al.^19^ previously documented that exposure to propylene glycol, present in most e-liquids, can induce ocular irritation, dryness, and glandular dysfunction.

Additionally, the reductions observed in macular flow density and inferior macular choroidal thickness after EC exposure – both statistically significant – may indicate a transient retinal hemodynamic alteration. Nicotine, present in most e-liquids used by our participants, has been associated with vasoconstriction, endothelial dysfunction, and oxidative stress at the microvascular level^20,21^. Experimental studies have shown that EC aerosols can alter the blood-retinal barrier, induce retinal cell apoptosis, and reduce capillary perfusion^21^. These mechanisms may explain the structural findings in our study, even after a single exposure, and support the hypothesis that chronic use could lead to cumulative damage to retinal vascularization. This opens a relevant line of research in ophthalmology, where the impact of ECs remains poorly explored.

### Limitations

This study has several limitations that should be considered when interpreting the results. First, the sample size was small, limiting statistical power and the generalizability of the findings. The lack of standardization in the type of device and liquid used, as well as the absence of quantification of nicotine concentration or aromatic additives, adds variability to the exposure. Furthermore, the pre–post design without a control group prevents direct causal inference. Finally, given the acute nature of the exposure, the findings cannot be extrapolated to repeated or chronic use scenarios. The results of this study suggest the need to investigate the long-term effects of vaping, particularly in adolescents and dual users. Future research should employ longitudinal designs with larger sample sizes and homogenous exposure groups.

## CONCLUSIONS

This study demonstrates that the acute use of electronic cigarettes can induce short-term measurable alterations at both the respiratory and ophthalmological levels post-exposure. Significant reductions in FeNO and increased pulmonary resistance were observed in dual users, suggesting an immediate inflammatory response of the respiratory epithelium. At the ocular level, changes were detected in tear production and retinal structural parameters, consistent with a transient hemodynamic disturbance.

These findings highlight the need to inform the public, particularly young individuals, about the potential risks associated with vaping, and underscore the importance of further research into its long-term effects through controlled studies with larger sample sizes.

## Data Availability

The data supporting this research are available from the authors on reasonable request.
